# *Dll1* haploinsufficiency causes brain abnormalities with functional relevance

**DOI:** 10.3389/fnins.2022.951418

**Published:** 2022-12-14

**Authors:** Dulce-María Arzate, Concepción Valencia, Marco-Antonio Dimas, Edwards Antonio-Cabrera, Emilio Domínguez-Salazar, Gilda Guerrero-Flores, Mariana Gutiérrez-Mariscal, Luis Covarrubias

**Affiliations:** ^1^Instituto de Biotecnología, Universidad Nacional Autónoma de México, Cuernavaca, Mexico; ^2^Departamento de Biología de la Reproducción, Universidad Autónoma Metropolitana Unidad Iztapalapa, Ciudad de México, Mexico

**Keywords:** notch signaling, neuronal density, microcephaly, hydrocephalus, myelination

## Abstract

**Introduction:**

The Notch pathway is fundamental for the generation of neurons during development. We previously reported that adult mice heterozygous for the null allele of the gene encoding the Delta-like ligand 1 for Notch (*Dll1^lacZ^*) have a reduced neuronal density in the substantia nigra pars compacta. The aim of the present work was to evaluate whether this alteration extends to other brain structures and the behavioral consequences of affected subjects.

**Methods:**

Brains of *Dll1*^+/lacZ^ embryos and mice at different ages were phenotypically compared against their wild type (WT) counterpart. Afterwards, brain histological analyses were performed followed by determinations of neural cell markers in tissue slices. Neurological deficits were diagnosed by applying different behavioral tests to *Dll1*^+/lacZ^ and WT mice.

**Results:**

Brain weight and size of *Dll1*^+/lacZ^ mice was significantly decreased compared with WT littermates (i.e., microcephaly), a phenotype detected early after birth. Interestingly, enlarged ventricles (i.e., hydrocephalus) was a common characteristic of brains of Dll1 haploinsufficient mice since early ages. At the cell level, general cell density and number of neurons in several brain regions, including the cortex and hippocampus, of *Dll1*^+/lacZ^ mice were reduced as compared with those regions of WT mice. Also, fewer neural stem cells were particularly found in the adult dentate gyrus of *Dll1*^+/lacZ^ mice but not in the subventricular zone. High myelination levels detected at early postnatal ages (P7–P24) were an additional penetrant phenotype in *Dll1*^+/lacZ^ mice, observation that was consistent with premature oligodendrocyte differentiation. After applying a set of behavioral tests, mild neurological alterations were detected that caused changes in motor behaviors and a deficit in object categorization.

**Discussion:**

Our observations suggest that Dll1 haploinsufficiency limits Notch signaling during brain development which, on one hand, leads to reduced brain cell density and causes microcephaly and hydrocephalus phenotypes and, on the other, alters the myelination process after birth. The severity of these defects could reach levels that affect normal brain function. Therefore, Dll1 haploinsufficiency is a risk factor that predisposes the brain to develop abnormalities with functional consequences.

## Highlights

- *Dll1* haploinsufficient mice (*Dll1*^+/lacZ^) develop microcephaly and hydrocephalus neuropathologies
- *Dll1*^+/lacZ^ mice showed a reduced neuronal density
- *Dll1*^+/lacZ^ mice have a reduced NSC pool in the hippocampus
- *Dll1*^+/lacZ^ mice showed an altered myelination pattern
- *Dll1*^+/lacZ^ mice showed neurological deficits
- *Dll1* haploinsufficiency is a risk factor for congenital brain malfunctions

## Introduction

The Notch pathway is highly conserved and fundamental for the generation of new neurons and their specification during development (Pierfelice et al., 2011). In mammals, Notch is a family of transmembrane receptors activated by transmembrane ligands such as Delta-like (DLL1, DLL3, and DLL4) and Jagged (JAG1 and JAG2) family members. After its activation, the Notch intracellular domain (NICD) is released and translocated to the nucleus, where it binds to RBPjk and induces the expression of genes such as members of the *Hes* and *Hey* gene families (Louvi and Artavanis-Tsakonas, 2006). The blockade of this pathway leads to neural precursor cells (NPCs) exhaustion by promoting their early differentiation, which results in a decrease in the number of neurons (Grandbarbe et al., 2003; Trujillo-Paredes et al., 2016). In the glial lineages, Notch signaling promotes astrocytic differentiation (Freeman, 2010) or oligodendrocyte precursor cell proliferation (Wang et al., 1998). Although the requirement of a specific Notch ligand is associated with a particular function, Delta-like canonical Notch ligand 1 (DLL1) appears to be the most widely distributed and more relevant both during development and in the adult (Beckers et al., 1999; Siebel and Lendahl, 2017).

We previously reported that mouse embryos homozygous for a *Dll1* null mutation (*Dll1^lacZ/lacZ^*) show premature dopaminergic differentiation in the midbrain, which is accompanied by a reduction in NPCs without altering their specification (Trujillo-Paredes et al., 2016). This phenomenon appears to be the main cause of the fewer dopaminergic neurons present in the substantia nigra pars compacta of adult mice heterozygous for the *Dll1^lacZ^* null allele (Trujillo-Paredes et al., 2016). Since *Dll1^lacZ/lacZ^* mice die few days after neurogenesis initiation (i.e., E11.5-E12.5; Hrabe de Angelis et al., 1997; Trujillo-Paredes et al., 2016), likely due to a disruption in vasculature development, detailed analysis of the role of DLL1 in the control of differentiation by Notch within different neuronal lineages is not possible.

Congenital anomalies can result from gene-environment interactions that modify the penetrance of defective alleles (Nishimura and Kurosawa, 2022). In particular, it is apparent that genes encoding Notch signaling components are prompt to environmental influences. In humans, members of families carrying putative mutant alleles for NOTCH1, NOTCH2, JAG1, DLL3, MESP2, LFNG, and HES7 show congenital anomalies of the heart and the vertebrae (Sparrow et al., 2012). Evidence of environmental influence has been demonstrated by showing that *Hes7* haploinsufficient mouse embryos exposed to hypoxia at certain developmental stage increases the incidence of scoliosis, a typical abnormality associated with Notch signaling deficiency (Sparrow et al., 2012). Since Notch signaling is key for the correct development of the brain, it is predicted that congenital brain anomalies develop under reduced Notch signaling.

Considering our previous observations (Trujillo-Paredes et al., 2016), the aim of the present work was to extend the analysis of brain abnormalities in *Dll1* haploinsufficient adult mice to underscore the possible congenital neurological dysfunctions that may emerge under reduced Notch signaling. The present work reveals distinct brain abnormalities (i.e., microcephaly, hydrocephalus, altered myelination) that might be the cause of brain function and mouse behavior alterations.

## Results

### Anatomical and histological evaluation

**FIGURE 1:**
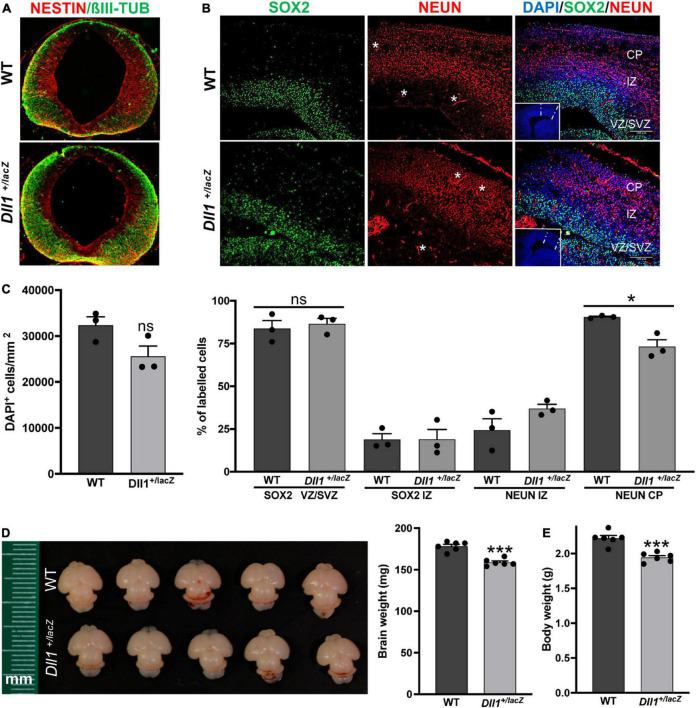
*Dll1* haploinsufficiency causes abnormalities in embryonic and neonatal brains. **(A)** Immunolabelling against NESTIN and βIII-TUBULIN in coronal mesencephalon sections of E11.5 WT and *Dll1*^+/lacZ^ embryos. A more abundant amount of βIII-TUBULIN and a reduced amount of NESTIN was a recurrent characteristic found in samples from *Dll1*^+/lacZ^ embryos when compared with WT embryos derived from the same pregnant female. **(B)** SOX2, NEUN (nuclear staining) and DAPI staining images in coronal telencephalon sections of E15.5 WT and *Dll1*^+/lacZ^ embryos; insets are low magnification images of the telencephalic sections under analysis (CP, cortical plate; IZ, intermediate zone; VZ, ventricular zone; SVZ, subventricular zone; asterisks, autofluorescence from blood vessels; scale bar, 100 μm). **(C)** Quantification of cell density (DAPI^+^ cells/mm^2^), and determination of the proportion of SOX2^+^ progenitors and NEUN^+^ neurons in samples as those shown in B (*P < 0.05; number of telencephalons analyzed = 3). Note that *Dll1* haploinsufficiency did not change the number of SOX2^+^ cells or neurons in the developing cortex, but their distribution was altered, resulting in a reduced number of neurons in the CP of samples from *Dll1*^+/lacZ^ embryos. **(D)** Representative brains of WT and *Dll1*^+/lacZ^ mice at postnatal day 3 (P3). **(E)** Brain and body weight of WT and *Dll1*^+/lacZ^ mice at P3 (***P < 0.001; n = 6 for each genotype). From these observations, it is apparent that *Dll1*^+/lacZ^ mice are born with a brain smaller than that of their WT siblings.

*Dll1*^+/lacZ^ embryos were largely normal, nonetheless, mild brain abnormalities were observed. These abnormalities could be related to signs of premature neurogenesis (i.e., more neurons and less NPCs), as seen in the mesencephalon of *Dll1*^+/lacZ^ embryos at E11.5 (Figure 1A; see also Trujillo-Paredes et al., 2016), whereas others might be due to a developmental asynchrony (i.e., altered progression from NPCs toward neurons), as it is apparent in the developing cortex of most *Dll1* haploinsufficient embryos compared with their WT littermates (Figures 1B,C). Allele segregation analysis of progeny from backcrosses suggests *Dll1^lacZ^* mendelian inheritance and low embryo lethality (46.2 ± 3.6% *Dll1*^+/lacZ^ born mice, *n* = 213). Interestingly, *Dll1*^+/lacZ^ newborn mice were smaller than age-matched WT mice, with a reduced total body and brain weight (Figures 1D,E). Without considering rare cases (less than 1 among 500 mice) in which *Dll1*^+/lacZ^ young mice (i.e., before 2 months of age) were sacrificed due to a spontaneous very high hyperactivity, most pups born carrying the *Dll1^lacZ^* allele survived to adulthood.

**FIGURE 2:**
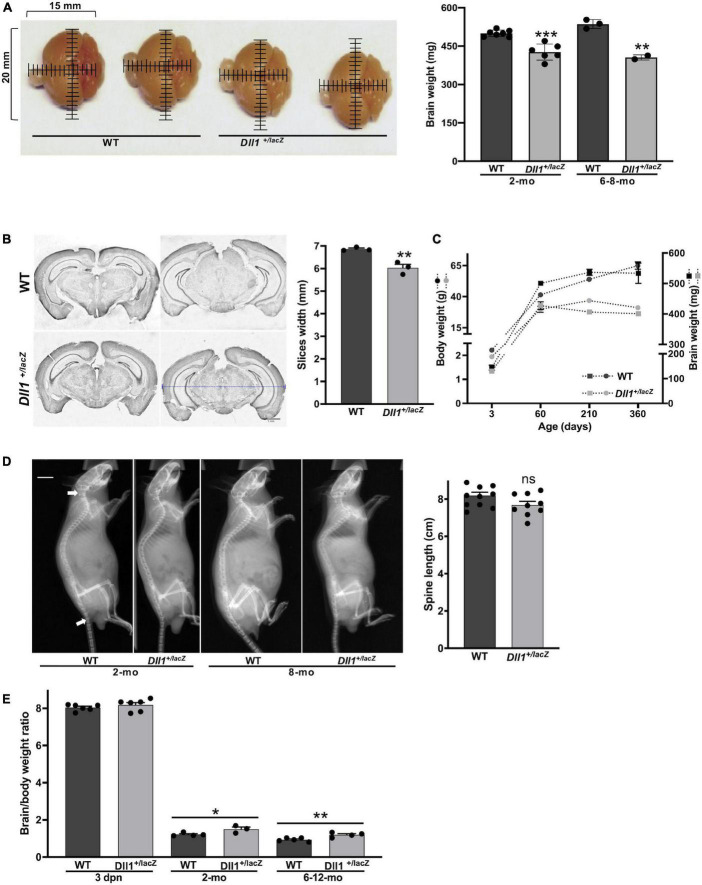
*DII1*^+laZ^ mice have smaller brains than WT mice. **(A)** Brains of WT and *Dll1*^+/lacZ^ adult mice. Images allow to appreciate the smaller brain size of *Dll1*^+/lacZ^ mice in comparison with that of WT mice (2 months of age; mm, millimeters), which is confirmed by comparing the weight of brains in graph (WT, *n* = 10, and *Dll1*^+/lacZ^, *n* = 8, of 2-months-old and 6-8-months-old mice; ***P < 0.001). **(B)** Representative coronal brain slices from WT and *Dll1*^+/lacZ^ adult mice after Nissl staining and the corresponding average width of fifteen rostrocaudal matched slices per subject (*n* = 3, **p < 0.01). The blue dashed line denotes the width measurement (scale bar, 1 mm). **(C)** Evolution of body (left) and brain (right) weight of WT and *Dll1*^+/lacZ^ mice along aging. **(D)** Spine length. Spine length was determined from X-ray skeletal images (left); arrows indicate the references used for measurements (WT, *n* = 11, and *Dll1*^+/lacZ^, *n* = 9; ns, no significant). **(E)** Brain to body weight ratio of WT and *Dll1*^+/lacZ^ animals (*P < 0.05 and **P < 0.01). All data together support that microcephaly is a phenotype of mice with reduced DLL1 levels.

Brains of adult *Dll1*^+/lacZ^ mice were smaller compared with brains of WT mice of the same age and strain (Figure 2A). This was consistent with measurements done on slices of brains of aged mice (Figure 2B). In agreement with a previous report (Rubio-Aliaga et al., 2009), a decline in body weight gain as aging progress was noted for *Dll1*^+/lacZ^ mice in comparison with WT mice (Figure 2C), but a significant difference was not noted in mouse size, as estimated by X-ray skeletal analysis (Figure 2D). Therefore, although the specific reduction in brain size of *Dll1*^+/lacZ^ mice was not apparent in newborn mice due to a comparative decrease in body weight (Figure 2C; see also Figures 1D,E), this phenotype became evident as mice aged (Figure 2E).

**FIGURE 3:**
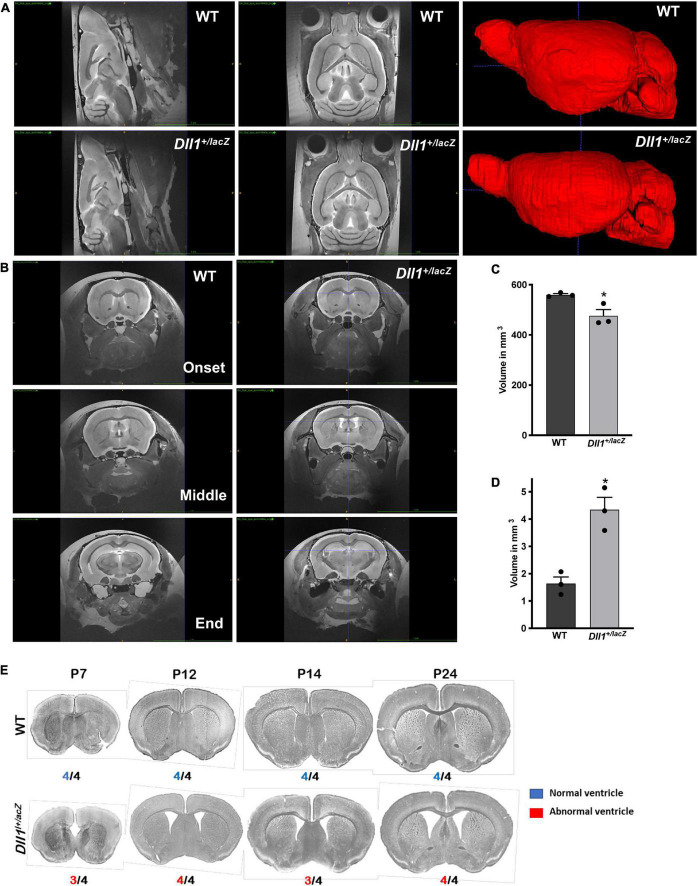
Increased lateral ventricle volume in brains of *Dll1*^+/lacZ^ mice. The volumes of whole brain and lateral ventricles were determined by MRI analysis. **(A)** Sagittal and dorsoventral views of representative brains of WT and *Dll1*^+/lacZ^ mice (4-5 months of age). On the right, 3D reconstructions after a manual segmentation of brains are shown. **(B)** Coronal views of brains of WT and *Dll1*^+/lacZ^ mice showing the initial, the middle and the final position along the rostro-caudal axis used to determine the lateral ventricle volume. **(C,D)** Total **(C)** and lateral ventricle **(D)** volume of brains of WT and *Dll1*^+/lacZ^ mice (*n* = 3 for both genotypes; Student *t*-Test: *P < 0.05). Note that, because the ventricular volume is included in the determinations of total brain volume, the microcephaly phenotype estimated from the total volume is an underestimation of the actual reduction in brain tissue of *Dll1*^+/lacZ^ mice. **(E)** Incidence of enlarged ventricles in *Dll1*^+/lacZ^ mice. Ventricular size was estimated from coronal slices of brains of WT and *Dll1*^+/lacZ^ mice at different postnatal ages (P7 to P24); incidence of the phenotype shown is indicated below each image. Note that the high incidence of enlarged ventricles was observed from a very early age after birth (P7) and remained up to reaching adulthood (after P24).

MRI analysis performed in adult brains confirmed the smaller size of the brain of *Dll1*^+/lacZ^ mice; total brain volume of *Dll1*^+/lacZ^ mice was about 15% less than that of WT mice (Figures 3A,C). Notably, the reduced brain volume of *Dll1*^+/lacZ^ mice included a larger ventricular volume in comparison with that present in brains of WT mice (Figures 3B,D, 5D,E). Suggesting a developmental origin, this latter characteristic was a penetrant phenotype noted from a few days after birth in brains of mice carrying the *Dll1^lacZ^* null allele and, interestingly, the ventricular volume increased as mice reached adulthood (Figure 3E).

### Cell density analysis

**FIGURE 4:**
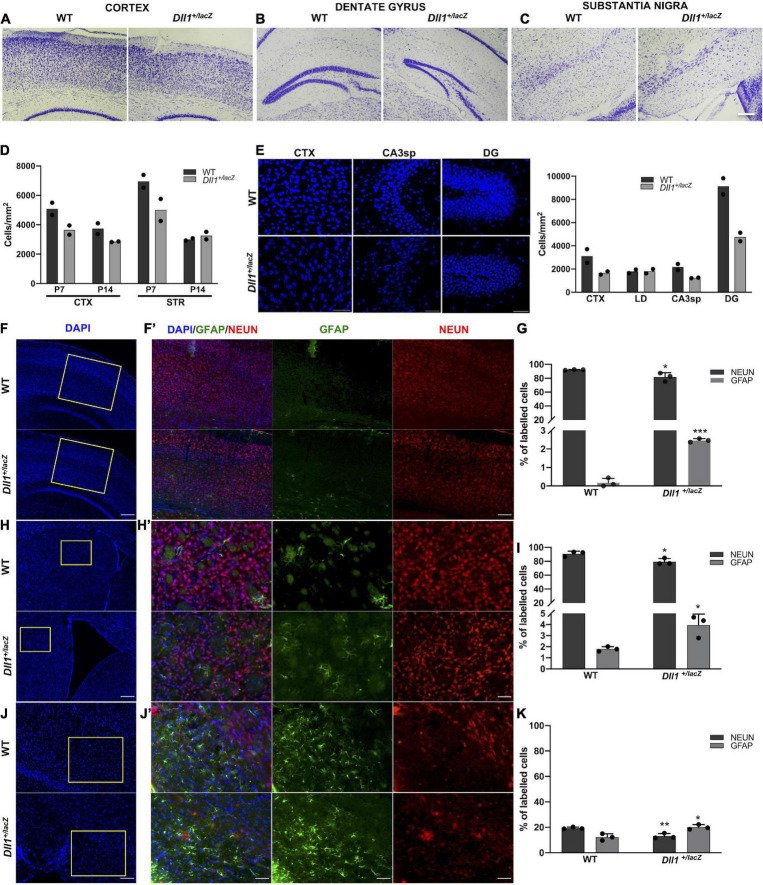
Adult brains of *Dll1*^+/lacZ^ mice have an altered density of neurons and astrocytes. **(A–C)** Representative images of the cortex, the dentate gyrus and the substantia nigra from brain slices stained with cresyl violet (Nissl staining; scale bar, 100 μm). **(D)** Cell density quantification in selected brain areas of postnatal mice (P7, P14). **(E)** Representative images of DAPI stained samples showing cell density in selected brain regions (CTX, cortex; STR, striatum; LD, lateral dorsal nucleus of thalamus; CA3sp, CA3 pyramidal layer; DG, dentate gyrus) of 3-months-old mice; the corresponding quantifications are shown in the graph. Observe the decreased cell density in samples from *Dll1*^+/lacZ^ mice in comparison with those from WT mice. **(F,H,J)** Panoramic views (nuclei counterstained with DAPI; scale bar, 200 μm) and the corresponding insets with GFAP^+^ and NEUN^+^ cells in the cortex (**F,F’**; scale bar 200 μm and 50 μm, respectively), the striatum (**H,H’**; scale bar 200 μm and 50 μm, respectively), and the substantia nigra pars reticulata (**J,J’**; scale bar, 100 μm and 50 μm, respectively) of *Dll1*^+/lacZ^ mice and their WT siblings are shown. Percentage of GFAP^+^ and NEUN^+^ cells, calculated from total DAPI^+^ cells in those regions, are shown in graphs **(G,I,K)**. Observe the reduction in the percentage of neurons and the increase in the proportion of glial cells in all regions shown (*n* = 3; *P < 0.05, **P < 0.01, ***P < 0.001).

In addition to the apparent reduction in the size of some structures (e.g., dentate gyrus, substantia nigra pars compacta) and in the thickness of cortex layers, brains of *Dll1*^+/lacZ^ mice also showed a more scattered distribution of cells in comparison with brains of WT mice (Figures 4A-C). The differences in cell density were not homogeneous, finding only trends to reduced cell density in some regions (e.g., striatum, substantia nigra pars reticulata) of brains of adult *Dll1*^+/lacZ^ mice in comparison with brains of WT mice (data not shown). Notably in the hippocampus, in addition to the apparent smaller size of the dentate gyrus (DG), lower cell density was found in the DG and the CA3sp areas and, in the cortex, the thinner layers correlated with a sustained reduction in cell density from early postnatal ages (Figures 4D,E).

As previously reported for the substantia nigra pars compacta, in which we found a reduction in the number of TH^+^ neurons (Trujillo-Paredes et al., 2016), a significant reduction in the percentage of NEUN^+^ cells in brains of *Dll1*^+/lacZ^ mice in comparison with those of WT mice was found in the cortex (Figures 4F,F’,G), the striatum (Figures 4H,H’,I) and the substantia nigra pars reticulata (Figures 4J,J’,K). Interestingly, the reduction in NEUN^+^ cells observed in these brain areas of *Dll1*^+/lacZ^ mice correlated with a corresponding increase in the percentage of GFAP^+^ cells (Figures 4G,I,K). These data suggest, not only a tendency to a reduction in brain cell density in *Dll1*^+/lacZ^ mice that correlates with a smaller brain size, but also a diminished proportion of neurons in the evaluated areas.

**FIGURE 5:**
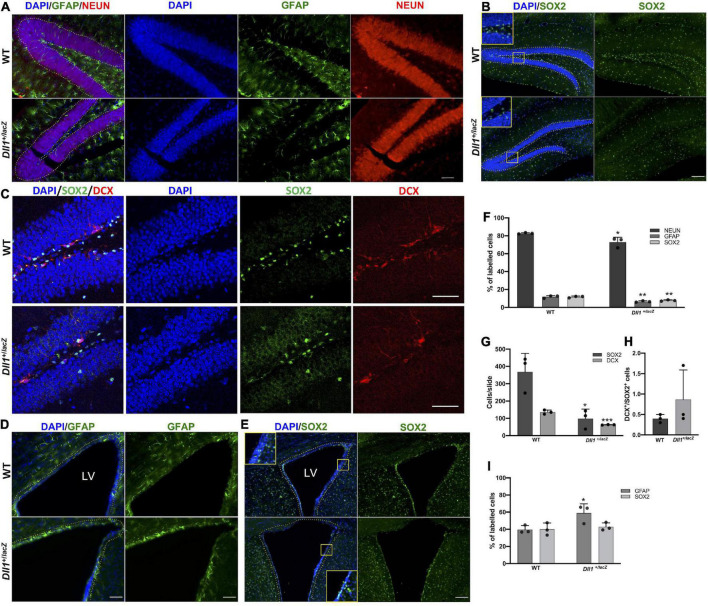
Neurogenic niches in the *Dll1*^+/lacZ^ mice have a reduced number of cells, including precursor cells. **(A–E)** Representative images showing GFAP^+^ and NEUN^+^ cells (**A**; scale bar, 50 μm), SOX2^+^ cells (**B,C**; scale bar, 100 μm), and DCX^+^ cells (**C**; scale bar, 50 μm) in the dentate gyrus; and GFAP^+^ (**D**; scale bar, 50 μm) and SOX2^+^ cells (**E**; scale bar, 100 μm) in the SVZ (LV, lateral ventricle). Quantifications of GFAP^+^, NEUN^+^, DCX^+^ and SOX2^+^ cells are shown in graphs (**F,G,I**; *n* = 3; *P < 0.05, **P < 0.005, ***P < 0.001). DCX^+^/SOX2^+^ cell ratio in the DG is also shown **(H)**. Only cells comprising the subgranular zone-granular cell layer or the cells lining the third ventricle were taken into account (dashed lines).

As Notch signaling participates in the generation of new neurons from two well-recognized neurogenic niches, the number of GFAP and SOX2 (a neural stem cell, NSC, marker) positive cells were determined in the DG of the hippocampus and the subventricular zone (SVZ). In agreement with a specific reduction in NSCs in the dentate gyrus, a lower percentage of GFAP^+^ cells (Figures 5A,F) and SOX2^+^ cells (Figures 5B,C,F,G) was found in the brain of *Dll1*^+/lacZ^ mice in comparison with that of WT mice, that was reflected in a reduction in the percentage of NEUN^+^ cells (Figures 5A,F). It was also found that there were fewer DCX^+^ neuroblasts in this region in association with *Dll1* haploinsufficiency (Figures 5C,G), but a significant difference was not found in the DCX^+^/SOX2^+^ cell ratio (Figure 5H), suggesting that adult neurogenesis is similarly active but limited by the number of NSCs present (see Discussion). In the SVZ of *Dll1*^+/lacZ^ animals in comparison with the WT counterpart, a significant reduction in the number of cells was evident (125.86 ± 7.0 vs. 227.33 ± 8.97 total cells within 20 μm from the ventricle/slide, *P* < 0.001; see insets in Figure 5E), which was accompanied by a significant increase in the proportion of GFAP^+^ cells (Figure 5D,I), but a significant difference was not found in the proportion of SOX2^+^ cells (Figures 5E,I).

### Myelination analysis

**FIGURE 6:**
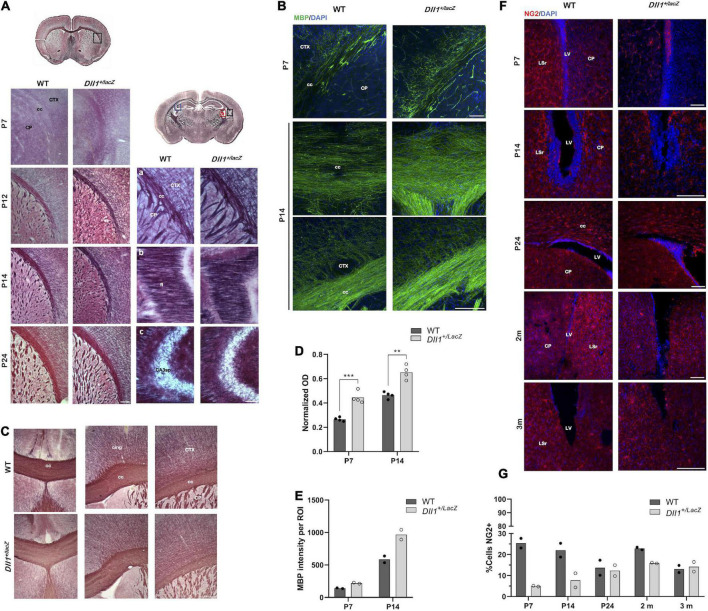
Myelination dynamics is altered in *DII1*^+/lacZ^ mice. **(A)** Areas (insets in drawns) of brain coronal slices from WT and *DII1*^+/lacZ^ mice at different postnatal ages (P7, P12, P14, P24) stained with Black-Gold II to reveal myelin levels (cc, corpus callosum; CTX, cortex; CP, caudo-putamen; fi, fimbria; *CA3sp*, pyramidal layer; scale bar, 100 μm). **(B)** Brain coronal slices, including similar areas as in A, from WT and *DII1*^+/lacZ^ mice at P7 and P14 stained for myelin basic protein (scale bar, 500 μm). Note that stainings were stronger in samples from *DII1*^+/lacZ^ mice than in those from WT mice. **(C)** Myelination levels in adult WT and *DII1*^+/lacZ^ mice. Images show Black-Gold II stained structures in equivalent brain slices of an anterior brain region of 2-months-old mice (cing, cingulum scale bar, 500 μm). Pictures are representative images from brain sections of a WT mouse, out of 7 analyzed, showing normal myelination levels (top panels) in comparison with images from sections of 1 brain found, out of 6 analyzed, of a *DII1*^+/lacZ^ mouse showing reduced myelination levels (bottom panels). **(D)** Quantification of normalized optical density (OD) of corpus callosum in images of samples stained with Black-Gold II (*n* = 4; **P < 0.01, ***P < 0.001). **(E)** Immunofluorescence intensity quantification of myelin basic protein in the corpus callosum of brains of mice at the postnatal ages indicated (*n* = 2). **(F)** Detection of NG2^+^ cells in areas of brain coronal slices from WT and *DII1*^+/lacZ^ mice at different postnatal ages (P7, P14, P24) and adult mice (2-3 months of age). Representative images are shown (LV, lateral ventricle; LSr, lateral septal nucleus rostral; scale bar, 100 μm). **(G)** Determinations of the percentage of NG2^+^ cells in the SVZ at different postnatal ages (P7, P14, P24) and of adult (2-3 months-old) mice. Note the marked increase in NG2^+^ cells in 2-months-old WT mice.

In contrast with WT mice, *Dll1*^+/lacZ^ mice showed increased levels of myelinated axons in most brain structures (e.g., corpus callosum, cortex, caudate putamen), from postnatal age P7 and up to P24, as assessed by Black-Gold II staining (Figures 6A,D) and detection of myelin basic protein (Figures 6B,E). This difference in myelination levels, however, was not detected in most 2-3-months-old adult mice (data not shown). Actually, only one among 5 brains of 2-3-months-old *Dll1*^+/lacZ^ mice showed reduced Black-Gold staining levels (Figure 6C) and myelin basic protein amount (data not shown) in distinct regions in comparison with 7 brains of WT mice. In contrast with the myelination pattern, fewer cells positive for NG2, a marker of oligodendrocyte precursor cells (OPCs), were found in different brain regions (e.g., corpus callosum, cortex, subventricular zone) of *Dll1*^+/lacZ^ mice than in those of WT mice from P7 and to P14 (Figure 6F). Specifically, in the subventricular zone, a neurogenic region that produces OPCs that migrate to different regions throughout life (Menn et al., 2006), a significant reduction in the number of NG2^+^ cells was observed (Figure 6G). By P24 no difference was noted in this region but, interestingly, in young adults (2-months-old mice), increased number of NG2^+^ cells was detected in the subventricular zone of WT mice but not in that of *Dll1*^+/lacZ^ mice, that later (in 3-months-old mice) decreased to equivalent levels for mice of both genotypes (Figures 6F,G). These observations are consistent with premature oligodendrocyte differentiation that leads to fewer oligodendrocyte precursors early in life and, later, to alterations that might impact the myelination dynamics as aging progresses.

### Neurological deficit evaluations

In order to determine general behavioral alterations in *Dll1* haploinsufficient mice, a set of neurological tests were performed (see Materials and methods; Tables 1, 2). A specific deficit was detected in hypomobility, piloerection and absence of equilibrium tests among young *Dll1*^+/lacZ^ male mice, and taking all neurological deficit tests together, young but not aged *Dll1*^+/lacZ^ mice showed a significant higher deficit score than WT male mice (Table 1). Male sexual behavior was generally normal (e.g., mounting and ejaculation per session), though it was noted that intromission in the first mating session was more frequent for young *Dll1*^+/lacZ^ mice than for WT mice (43% vs. 20%, respectively) and reached over 50% by the second and third sessions (Table 2).

**TABLE 1:** Neurological deficit evaluation.

Parameters	Young WT		Young *Dll1*^+/lacZ^		Aged WT		Aged *Dll1*^+/lacZ^	
**Phase 1**		*n*		*n*		*n*		*n*
Hypomobility	1.3 ± 0.1	*11/11*	1.7 ± 0.1*	*15/15*	2.0 ± 0.0	*3/3*	1.0 ± 0.0	*3/3*
Lateralized posture	0.0 ± 0.0	*0/11*	0.0 ± 0.0	*0/15*	0.0 ± 0.0	*0/3*	0.0 ± 0.0	*0/3*
Flattened posture	0.0 ± 0.0	*0/11*	0.0 ± 0.0	*0/15*	0.0 ± 0.0	*0/3*	0.0 ± 0.0	*0/3*
Hunched back	0.0 ± 0.0	*0/11*	0.0 ± 0.0	*0/15*	0.0 ± 0.0	*0/3*	0.0 ± 0.0	*0/3*
Piloerection	0.3 ± 0.1	*3/11*	0.8 ± 0.1*	*12/15^°^*	1.0 ± 0.0	*3/3*	0.7 ± 0.3	*2/3*
Ataxic gait	0.0 ± 0.0	*0/11*	0.0 ± 0.0	*0/15*	0.0 ± 0.0	*0/3*	0.0 ± 0.0	*0/3*
Circling	0.0 ± 0.0	*0/11*	0.0 ± 0.0	*0/15*	0.0 ± 0.0	*0/3*	0.0 ± 0.0	*0/3*
Tremors	0.2 ± 0.1	*2/11*	0.2 ± 0.1	*3/15*	0.3 ± 0.3	*1/3*	0.3 ± 0.3	*1/3*
Twitches	0.0 ± 0.0	*0/11*	0.1 ± 0.1	*1/15*	0.0 ± 0.0	*0/3*	0.0 ± 0.0	*0/3*
Convulsions	0.0 ± 0.0	*0/11*	0.0 ± 0.0	*0/15*	0.0 ± 0.0	*0/3*	0.0 ± 0.0	*0/3*
Respiratory distress	0.1 ± 0.1	*1/11*	0.0 ± 0.0	*0/15*	0.0 ± 0.0	*0/3*	0.3 ± 0.3	*1/3*
**Phase 2**								
Passivity	0.5 ± 0.2	*5/11*	0.5 ± 0.1	*8/15*	1.0 ± 0.0	*3/3*	0.7 ± 0.3	*2/3*
Hyperreactivity	0.0 ± 0.0	*0/11*	0.1 ± 0.1	*2/15*	0.0 ± 0.0	*0/3*	0.0 ± 0.0	*0/3*
Irritability	0.0 ± 0.0	*0/11*	0.1 ± 0.1	*2/15*	0.0 ± 0.0	*0/3*	0.0 ± 0.0	*0/3*
Ptosis	0.0 ± 0.0	*0/11*	0.0 ± 0.0	*0/15*	0.3 ± 0.3	*1/3*	0.0 ± 0.0	*0/3*
Urination	0.5 ± 0.2	*5/11*	0.1 ± 0.1	*2/15*	0.3 ± 0.3	*1/3*	0.0 ± 0.0	*0/3*
Decreased body tone	0.1 ± 0.1	*1/11*	0.3 ± 0.1	*5/15*	0.3 ± 0.3	*1/3*	0.3 ± 0.3	*1/3*
Forelimb flexion	0.1 ± 0.1	*1/11*	0.0 ± 0.0	*0/15*	0.0 ± 0.0	*0/3*	0.0 ± 0.0	*0/3*
Decreased muscle strength	0.1 ± 0.1	*1/11*	0.1 ± 0.1	*1/15*	0.7 ± 0.3	*2/3*	0.0 ± 0.0	*0/3*
Body rotation	1.2 ± 0.1	*11/11*	0.2 ± 0.1*	*3/15*°	0.3 ± 0.3	*1/3*	0.0 ± 0.0	*0/3*
Motor incordination	1.2 ± 0.1	*11/11*	1.5 ± 0.1	*15/15*	1.7 ± 0.3	*3/3*	2.3 ± 0.7	*3/3*
Absence of equilibrium	1.2 ± 0.1	*11/11*	1.8 ± 0.2*	*15/15*	2.0 ± 0.0	*3/3*	2.0 ± 0.6	*3/3*
Hypoalgesia	0.3 ± 0.1	*3/11*	0.6 ± 0.1	*9/15*	0.0 ± 0.0	*0/3*	0.3 ± 0.3	*1/3*
Hyperalgesia	0.0 ± 0.0	*0/11*	0.0 ± 0.0	*0/15*	0.0 ± 0.0	*0/3*	0.0 ± 0.0	*0/3*
**Phase 3**								
Vibrissae sensitivity	0.0 ± 0.0	*0/11*	0.0 ± 0.0	*0/15*	0.0 ± 0.0	*0/3*	0.0 ± 0.0	*0/3*
Touch sensitivity	0.0 ± 0.0	*0/11*	0.0 ± 0.0	*0/15*	0.0 ± 0.0	*0/3*	0.0 ± 0.0	*0/3*
Visual sensitivity	0.0 ± 0.0	*0/11*	0.0 ± 0.0	*0/15*	0.0 ± 0.0	*0/3*	0.0 ± 0.0	*0/3*
Olfactory sensitivity	1.6 ± 0.2	*11/11*	1.7 ± 0.2	*15/15*	1.3 ± 0.3	*3/3*	2.0 ± 0.5	*3/3*
Global score	7.5 ± 0.8		9.9 ± 0.6*		11.3 ± 1.2		10.0 ± 1.2	

Student-*t* test. *Different to Young WT group: *p* < 0.05. Fisher Exact test. Different to Young WT group: *p* < 0.05.

**TABLE 2:** Temporal (seconds) and numeric parameters of the copulatory behavior in young and aged WT and *Dll1*^+/lacZ^ male mice (ML, mounting latency; IL, intromission latency; EL, ejaculation latency; NM, number of mounts; NI, number of intromissions).

		Sessions					
	Group	1	*n*	2	*N*	3	*n*
ML	Young WT	1700.7 ± 218.9	*7/10*	944.4 ± 179.7	*8/10*	790.4 ± 163.2	*9/10*
	Young *Dll1*^+/lacZ^	1054.3 ± 75.0	*8/14*	1294.2 ± 181.1	*11/13*	1026.3 ± 196.4	*10/10*
	Aged WT	1871.0	*1/3*	1741.0 ± 59.0	*2/3*	—	*0/3*
	Aged *Dll1*^+/lacZ^	2329.0	*1/3*	—	*0/3*	—	*0/3*
IL	Young WT	1785.0 ± 315.0	*2/10*	1058.7 ± 166.4	*8/10*	863.0 ± 171.7	*9/10*
	Young *Dll1*^+/lacZ^	1345.0 ± 196.7	*6/14*	1522.6 ± 249.2	*7/13*	1164.6 ± 243.8	*9/13*
	Aged WT	—	*0/3*	—	*0/3*	—	*0/3*
	Aged *Dll1*^+/lacZ^	2329.0	*1/3*	—	*0/3*	—	*0/3*
EL	Young WT	—	*0/10*	—	*0/10*	2370.0	*1/10*
	Young *Dll1*^+/lacZ^	—	*0/14*	—	*0/13*	1759.0 ± 72.0	*2/13*
	Aged WT	—	*0/3*	—	*0/3*	—	*0/3*
	Aged *Dll1*^+/lacZ^	—	*0/3*	—	*0/3*	—	*0/3*
NM	Young WT	4.6 ± 1.6	*7/10*	11.1 ± 1.8	*8/10*	7.8 ± 1.8	*9/10*
	Young *Dll1*^+/lacZ^	8.1 ± 1.3	*8/14*	7.3 ± 1.3	*11/13*	10.7 ± 3.0	*10/13*
	Aged WT	3.0	*1/3*	2.0	*2/3*	—	*0/3*
	Old *Dll1*^+/lacZ^	2.0	*1/3*	—	*0/3*	—	*0/3*
NI	Young WT	6.0 ± 3.0	*2/10*	16.1 ± 4.0	*8/10*	12.4 ± 2.7	*9/10*
	Young *Dll1*^+/lacZ^	4.8 ± 1.4	*6/14*	7.9 ± 1.6	*7/13*	9.1 ± 2.7	*9/13*
	Aged WT	—	*0/3*	—	*0/3*	—	*0/3*
	Aged *Dll1*^+/lacZ^	—	*1/3*	—	*0/3*	—	*0/3*

**FIGURE 7:**
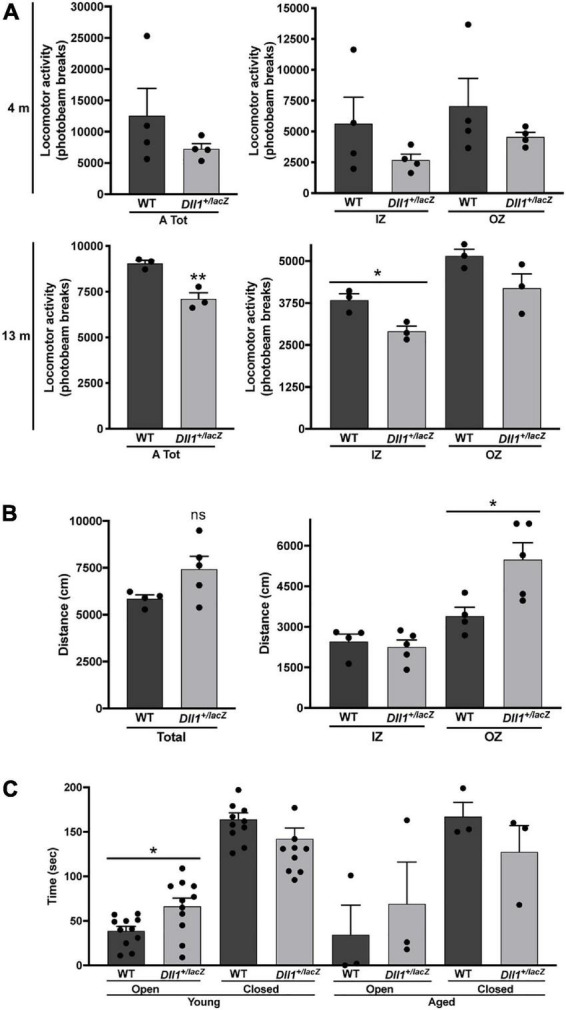
*Dll1* haploinsufficiency leads to locomotor behavioral alterations. **(A)** Mean locomotor (ambulatory) activity recorded as total (left graph) or inner (IZ) and outer (OZ) zone (right graph) photobeam breaks for 12 dark hours (see Materials and Methods) of WT and *Dll1*^+/lacZ^ mice; young (4 months-old; *n* = 4 of each genotype) and aged (13 months-old; *n* = 3 of each genotype) mice were analyzed (**P < 0.01; *P < 0.05). **(B)** Total distance traveled (left graph) and distance traveled in the IZ or OZ (right graph) by young WT (*n* = 4) and *Dll1*^+/lacZ^ (*n* = 5) mice (*P < 0.05). Note that, although contrasting differences between *Dll1*^+/lacZ^ and WT mice were observed in the locomotor activity tests performed, likely, each test was revealing a distinct behavioral deficit. **(C)** Time spent in the open or closed arms of the elevated plus maze by WT (*n* = 11) or *Dll1*^+/lacZ^ (*n* = 11) mice of 4-5 months of age (young) and WT (*n* = 3) or *Dll1*^+/lacZ^ (*n* = 3) mice of 14-16 months of age (aged) (*p < 0.05).

Alterations in locomotor activity were assessed in more detail. Using a laser-based detector of locomotor activity (see Materials and methods; Figure 7A) the accumulative ambulatory movements of mice after habituation were recorded along the natural activity period (i.e., 12 h of darkness). Although it was only apparent at young adult age that *Dll1*^+/lacZ^ mice moved less (i.e., fewer beam breaks) than WT mice, aged mice showed a significant difference in this test (Figure 7A), more evident within the inner zone (Figure 7A). In a different test, ambulatory movements of young adults in an unexplored area were determined using a video-based detector within a 5 min time limit (Figure 7B). Interestingly, in this latter test, *Dll1*^+/lacZ^ mice moved a longer distance than WT mice, activity that occurred mostly in the outer zone (Figure 7B). As time in the outer versus the inner zone was not significantly different (data not shown), this behavior cannot be considered a sign of increased anxiety. Accordingly, in the elevated plus maze test, despite that all mice stayed most of the time in the closed arm, *Dll1*^+/lacZ^ mice spent more time in the open arm than WT mice soon after exposure to a novel environment (Figure 7C). Therefore, it is apparent that *Dll1* haploinsufficient mice move less in the long term but, in the short term, a hyperactive-like behavior is uncovered under certain circumstances (e.g., soon after exposed to a novel environment). In agreement with this conclusion, a previous study reports hyperactivity in *Dll1* haploinsufficient mice under a different genetic background than the one of mice used here (Rubio-Aliaga et al., 2009).

**FIGURE 8:**
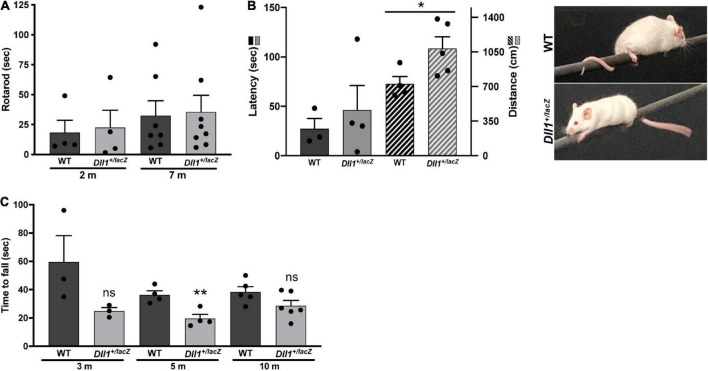
Differences in motor activity and strength performance due to *Dll1* haploinsufficiency. WT and *Dll1*^+/lacZ^ mice were tested for motor functions. **(A)** Rotarod test. Two- (n = 4 of each genotype) and 7-months-old (*n* = 7, WT and *n* = 8, *Dll1*^+/lacZ^) mice were used. Due to the high variability of this test, no difference between WT and *Dll1*^+/lacZ^ mice was observed. **(B)** Balance beam test. WT (*n* = 3) and *Dll1*^+/lacZ^ (*n* = 4) young mice were used; note that, although there was no significant difference in the time to fall from the bar, *Dll1*^+/lacZ^ mice traveled a longer distance on the bar than WT mice (*P < 0.05). Images show how mice used its tail to stay on the bar. **(C)** Inverted grid test. Mice at 3 different ages were tested: 3 months-old (WT, n = 3; *Dll1*^+/lacZ^, *n* = 3), 5 months-old (WT, n = 4; *Dll1*^+/lacZ^, n = 4), and 10 months-old (WT, n = 5; *Dll1*^+/lacZ^, *n* = 6). Generally, a decreased motor strength was detected in *Dll1*^+/lacZ^ mice, though significance was limited (**P < 0.01).

The neuromuscular function was evaluated by performing equilibrium and gripping tests (Figure 8). No major differences in performance by *Dll1*^+/lacZ^ and WT mice was observed in the rotarod test (Figure 8A). In addition, performance differences in the equilibrium bar test were not found (Figure 8B). However, it was noted that most *Dll1*^+/lacZ^ mice did not wrap their tail on bar and traveled a longer distance than WT mice (Figure 8B), possibly reflecting a neurological deficit, but it cannot be discarded that it is due to tail vertebra abnormalities, frequent in *Dll1* haploinsufficient mice (Rubio-Aliaga et al., 2009). In contrast, the hanging time of Dll1^+/lacZ^ mice was significantly shorter than the time WT mice remained suspended (in 5-months-old mice but only apparent in 3- and 10-months-old mice; Figure 8C). Therefore, these tests did not reveal motor coordination abnormalities, but gripping strength appeared reduced in *Dll1* haploinsufficient mice.

**FIGURE 9:**
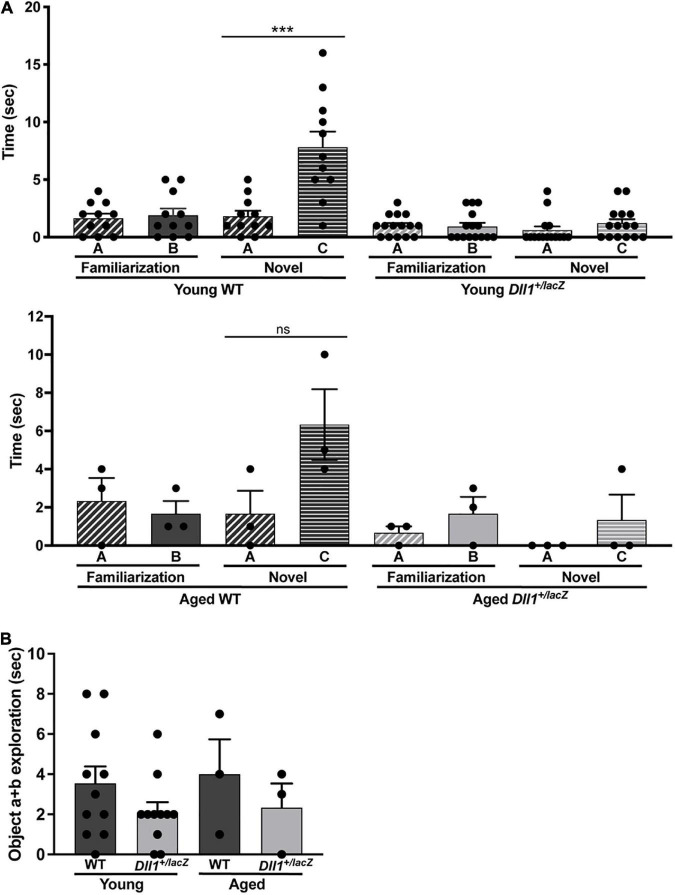
*Dll1*^+/lacZ^ male mice have a deficit in the ability to categorize objects. Young WT (*n* = 11) and *Dll1*^+/lacZ^ (*n* = 11) or aged WT (*n* = 3) and *Dll1*^+/lacZ^ (*n* = 3) male mice were used for the test. The time mice spent with A or B objects during the familiarization session and the time spent exploring A or C objects in the novel session were evaluated **(A)**. Additionally, we show the time spent by mice exploring any object during the familiarization session **(B)**. Note that, in the novel session, *Dll1*^+/lacZ^ mice did not show the typical increase in time spent exploring the novel object (***p < 0.05 for young mice; ns, no statistically significant). In addition to the impairment in novel object recognition, *Dll1*^+/lacZ^ mice also showed an apparent reduction in total time devoted to object exploration (ns, no statistically significant).

A deficit in object recognition was a highly penetrant phenotype due to *Dll1* haploinsufficiency. As expected, WT mice spent a longer time exploring a novel object than the familiar one but, in contrast, *Dll1*^+/lacZ^ mice spent the same time exploring both objects (Figure 9A). These observations are in agreement with a deficit in the ability to identify objects (i.e., deficit in recognition memory). As estimated from comparing the immobility time of mice in the tail suspension test (data not shown), no depressive behavior was detected due to *Dll1* haploinsufficiency, but the apparent shorter exploration time devoted to objects by *Dll1*^+/lacZ^ mice in comparison with WT mice (Figure 9B) suggests a cognitive disability (e.g., inability to categorize objects).

## Materials and methods

### Animals

All experiments were performed under the ethical guidelines of the Instituto de Biotecnologia, UNAM and of the Universidad Autónoma Metropolitana-Unidad Iztapalapa. In the mice studied, the *DIl1^lacZ^* allele is under the outbred genetic background of the CD1 strain. Mice were genotyped by staining for LacZ and/or by an allele-specific PCR. Backcrosses were performed in order to maintain the genetic variability of the CD1 strain, that is, matings between brothers and sisters or parents were avoided, and non-related WT CD1 mice were introduced after 3-4 matings. Generally, male mice were studied, and for comparisons, divided into groups by age. The animals were housed in a room with a light-dark cycle of 12-12h and controlled temperature of 21 ± 1°C. The water and food were provided *ad libitum*. Mice were sacrificed by cervical dislocation or by an intraperitoneal injection of a pentobarbital overdose (200 mg/Kg pentobarbital/mouse or 20 μl/g 2.5% avertin). When required, a perfusion was intracardially performed, first with 30 ml of phosphate buffer saline (PBS 0.1M) at 100 ml/h, and then with 30 ml of 4% paraformaldehyde (PFA) at the same rate. Specifically for MRI acquisitions, six young WT and *Dll1*^+/lacZ^ mice were used and the PFA solution was supplemented with gadolinium (2 mM, Gadovis kindly provided by Prof. Andres Morón, Centro Nacional de Imagenología e Instrumentación Médica, Universidad Autónoma Metropolitana). The heads were removed along with the skin, lower jaw, ears, and the cartilaginous nose tip. The skull was then stored in PBS 0.1M containing sodium azide (0.02%) and gadolinium (2 mM) at 15°C for, at least, four days and not longer than 2.5 months post-perfusion before MRI acquisition (see below).

### Anatomical and histological evaluations

Brains of animals were analyzed at different ages. Brains were collected and cryopreserved before obtaining 10-50 μm slices, which were preserved at −70°C until use. Brains of E11.5-E15.5 embryos were fixed for 2 h at 4°C in 4% PFA in PBS, washed with PBS, dehydrated with 30% sucrose and sectioned (10 μm slices) after freezing (Leica cryostate CM 1850, Wetzlar, Germany). P7-P24 and adult mice were perfused intracardially, first with PBS, followed by 4% PFA. Brains were removed and placed in 4% PFA for 24 h. Subsequently, the tissues were cryopreserved by immersion in 30% sucrose for at least 48 h; 50 μm (early postnatal ages) or 30 μm (adult) slices were obtained. Nissl and DAPI staining was used to evaluate cell distribution in tissue slices. Immunostaining for embryonic brain and adult brain sections was performed as previously described in Guerrero-Flores et al. (2017) and in Arzate et al. (2019), respectively. For cell density quantifications, only cells comprised within the region under evaluation were taken into account. Primary antibodies used were: anti-Nestin (Millipore, MAB353), anti-SOX2 (Santa Cruz, sc-17320), anti-NEUN (Chemicon, MAB377), anti-GFAP (DAKO, Z0334), anti-DCX (Santa Cruz, sc-8067), anti-MBP (Covance, SMI-99) and anti-NG2 (Millipore, AB5320). Secondary antibodies used were Alexa 488- and Alexa 595-conjugate anti-mouse, anti-goat or anti-rabbit antibodies (Invitrogen). After single or double staining, sections were mounted and analyzed using a Zeiss Apotome microscope (Zeiss, Oberkochen, Germany) and a confocal microscope Olympus FV1000 (Olympus, Tokyo, Japan).

### Myelination analysis

Brain frozen sections (50 μm thick) mounted on slide were stained using the Black-Gold II Myelin Staining Kit (Histo-Chem Inc., #2BGII). Briefly, samples were incubated with the Black-Gold II solution for up to 15 min at 65°C, washed twice with water, incubated with 1% sodium thiosulfate solution for 3 min at 65°C, and washed three times with water before dehydration and mounting. Myelination levels were quantified by determining the mean optical intensity of a delimited area (region of interest, ROI; i.e., corpus callosum) in grayscale images (8 bits) of stained sections using the FIJI Image J. software. The normalized optical density values were calculated by substracting the mean optical intensity of the ROI from the intensity detected in the background of each image and then divided by the same factor. Fluorescence intensity after MBP immunofluorescence was also quantified using the FIJI Image J. software applied on confocal images. In this case the ROI in images was delimited (corpus callosum) and the intensity calculated by substracting the background intensity from the mean intensity in the ROI.

### X-ray and magnetic resonance image analysis

Young and adult WT and *Dll1*^+/lacZ^ mice were anesthetized and X-ray images captured using the *In vivo* Xtreme instrument (Bruker, Billerica, Massachusetts). The mice spine length was estimated based on X-ray images considering the distance from neck to tail base, (WT, *n* = 11; *Dll1*^+/lacZ^, *n* = 9). MRI acquisition was conducted with a Bruker Pharmascan 70/16US, 7 Tesla magnetic resonance scanner (Bruker, Ettlingen, Germany). An anatomical scan was obtained using a spin-echo rapid acquisition with refocused echoes (Turbo-RARE) sequence with the following parameters: TR = 400 ms, TE = 21.3 ms, matrix dimensions = 396 × 396, 396 slices, slice thickness = 0.040 mm, no gap, resulting in isometric voxels 0.040 × 0.040 × 0.040 mm^3^ in size. The volume of the whole brain and lateral ventricles were determined manually drawing regions of interest (ROIs) using the segmentation function of ITK-SNAP software (Yushkevich et al., 2006). For LV measurements, we considered the volume from the anterior truncated part of the corpus callosum named the genu (onset in Figure 3B) to the splenium (end in Figure 3B).

### Behavioral evaluations

#### General neurological deficit evaluation

The neurological deficit evaluation was performed in a lighted room. We followed the protocols validated by Rodriguez et al. (2005) and Irwin (1968). In the first stage, the examiner recorded the presence or absence of a lateralized posture, flattened posture, hunched back, piloerection, ataxic gait, circling, tremors, twitches, convulsions, respiratory distress, and the degree of hypomobility (1 slight, 2 moderate, 3 severe). In the second stage with manipulation, the examiner recorded the passivity, hyperreactivity, irritability, ptosis, urination, decreased body tone, forelimb flexion, decreased muscle strength, body rotation, motor incoordination, absence of equilibrium, hypoalgesia, and hyperalgesia. Table 1 shows the average score determined for the number of mice indicated at two ages (young and aged).

#### Sexual behavior test

The male sexual behavior test was performed 2 h after the onset of darkness and under dim red light. Inside of the home-cage of each male mouse, a receptive female of the same strain (20–30 g) was introduced and allowed mating for 45 min or until ejaculation. The female mice were brought into estrous by hormone treatment. Specifically, mice were ovariectomized (GDX) and subcutaneously injected with 20 μg of estradiol benzoate (EB) and 500 μg of progesterone (P) 48 and 6 h, respectively, before each mating test. All male mice were evaluated in three mating sessions, each separated by at least 48 h. The number of male mice that mounted, intromitted or ejaculated is shown in Table 2, as well as, the latency of mount (LM; time from introduction of the male into the test cage until the first mount with pelvic thrusting), latency of intromission (LI; time for the first intromission behavior pattern), latency of ejaculation (LE; time from the first intromission behavior pattern until the first ejaculation behavior pattern), the number of mounts (NM) and the number of intromissions (NI) until ejaculation.

#### Locomotor activity tests

(a) Photobeam breaks Open Field test. Locomotor activity was tracked with the Photobeam Activity System-Home Cage (PAS-HC, 4 × 8 photobeam configuration; SD Instruments). Each mouse was habituated by placing the cage on the chamber for 4 h one day before the test. The test was run with one mouse per chamber for 24 h and photobeam breaks registered at 30 min intervals. Only movements during the dark phase were considered (20-48 intervals). (b) Video-tracking in an open field test. In a lighted room, animals were placed on the center of a 60 × 60 cm dark arena. Movements were recorded for 5 min using the SMART 2.0 software (PanLab, S.L.U. Barcelona, Spain); outer and inner zones were pre-defined. The software analyzes the trajectory of the animal and measures the time spent and the distance traveled in each defined zone.

#### Rotarod test

The Rotarod 755 unit was used (IITC Life Science, San Diego, CA. USA). Mice were habituated to the unit without movement for 1 min before running every trial, and trained by performing 2 sessions per day for 3 days at 4-20 rpm rate. The test included 5 consecutive determinations, one per day at the same daytime (in a lighted room). In the testing trials, the animals were placed at an initial speed of 10 rpm, and then rod speed gradually increased to 45 rpm over 180 s. Time and speed were registered until animal falls.

#### Inverted grid test

In a lighted room, mice were placed one by one on a grid (i.e., a cage lid), and after 5 s, the grid was inverted and held at 35 cm over a mouse cage with enough mouse bedding. The average time to fall (max 5 min) was recorded in 3 trials per session and 3 sessions along 3 consecutive days.

#### Elevated plus maze test

The elevated plus maze test was performed in a dark room with the elevated plus maze illuminated. The male mice were placed in the core of the elevated plus maze and were allowed to freely explore the arms for 5 min. The time that mice spent in the open or closed arm was registered.

#### Tail suspension test

The tail suspension test was performed 2 h after onset of darkness and under dim red light. Each mouse was suspended 40 cm up the floor by the tail with a masking tape. The immobility time was recorded in a 5 min session.

#### Object recognition test

The object recognition test (Walf et al., 2008; Leger et al., 2013) was performed in a lighted room. First, male mice were habituated to the open field box (60 × 40 × 40 cm) for 5 min once a day for 3 days. After the habituation sessions, each mouse was free to explore the two objects, different in form and texture, during 3 min (familiarization session). The time that each mouse spent exploring the objects was registered. Thirty minutes later, one object was replaced for a different one and the time that mice spent exploring this novel object vs. the familiar object (A vs. C) was registered (novel session).

#### Balance beam test

Each mouse, WT or *Dll1*^+/lacZ^, was individually placed at one end of a bar (1.0 m long, 1.0 cm in diameter, suspended at 60 cm height) and allowed to move along it for 2 min. Latency (time before first fall), number of falls and distance traveled were recorded using the SMART 2.5 software (PanLab, S.L.U. Barcelona, Spain). Two independent experiments were performed.

### Statistics

For statistical analysis, a two-tailed Student *t test* or One-Way ANOVA were employed for comparison between genotype conditions, except for data that did not pass a normality (Shapiro-Wilk) and the equal variance tests, in which case, the Rank sum Test followed by the Dunn’s method for multiple comparisons were employed. The data of copulatory parameters between test sessions were analyzed using the Analysis of Variance on Rank (Kruskal Wallis) followed by Dunn’s method for multiple comparisons. The percentage of male mice that displayed mounts, intromissions, and ejaculations in each sexual behavior test were analyzed using a Chi-square test. All values are represented as mean ± SEM. A P value less than 0.05 was considered significant.

## Discussion

Notch signaling participates in the differentiation of many cell types. Thus, a profound reduction in Notch signaling during development would be generally lethal, though in some instances, likely due to a transient Notch signaling depletion, animals survive but still display abnormalities in organs or structures (e.g., vertebrae, heart; Siebel and Lendahl, 2017). Mild reduction in Notch signaling apparently does not cause a major alteration in most tissues and organs, but the brain is expected to be more prone to morphological alterations with functional consequences. The observations of the present study are consistent with a major and non-redundant role of DLL1 in the regulation of Notch signaling during brain development, participating in the control of both neurogenesis and oligodendrogenesis. Therefore, *Dll1* haploinsufficiency in mice could be a useful genetic condition to determine non-life threatening abnormalities associated with Notch signaling that might influence brain function. The present study was not designed to identify the specific circuits affected by the shortage in Notch signaling but, rather, to uncover brain abnormalities that could cause neurological anomalies. We recognize that, perhaps due to the relatively small number of animals used for the determinations, a statistical difference was not observed in some instances. The recurrence of neurological anomalies, however, is evidence of the broad influence of Notch signaling in brain development and function and coincident with the expected incidence of a congenital disorder. Therefore, without referring to a specific brain function, *Dll1* haploinsufficiency can be considered a risk factor for congenital brain malfunctions.

### *Dll1* haploinsufficiency alters brain development

The brain alterations observed likely have a developmental origin. It is well known that reducing Notch signaling causes premature differentiation of stem cells within different lineages (Koch et al., 2013). In particular, reduction in Notch signaling causes neuronal differentiation of NPCs that, in some instances, is accompanied by an increase in astrocytes (Louvi and Artavanis-Tsakonas, 2006). The key role of Notch signaling in cell differentiation is the expansion of the stem cell population, upon which the number of cellular derivatives depends. Therefore, premature neuronal differentiation could cause an exhaustion of stem cells with the consequential reduction in the total number of neurons produced (Chapouton et al., 2010; Imayoshi et al., 2010; Trujillo-Paredes et al., 2016). Generally, signaling by Notch receptors depends on its recognized ligands, among which DLL1 is the most widely active. Considering that most neurons are produced before birth, *Dll1* haploinsufficiency could cause a mild premature neuronal differentiation in the embryo and, consequently, the reduction in the number of neurons observed in the adult brain. In agreement with a broad influence of DLL1 in the determination of neuronal density in the brain, mild microcephaly was a penetrant phenotype detected early after birth in *Dll1*^+/lacZ^ mice. Nonetheless, variations in the level of influence within different brain regions could be due to the participation of other Notch ligands or a compensation by additional factors in the niche controlling stem cell proliferation and maintenance (Fuchs and Blau, 2020).

Despite that Notch signaling in the adult brain neurogenic niches has a similar role in neuronal differentiation as during development (Imayoshi and Kageyama, 2011), the presence of other ligands should be considered to estimate how a decrease in DLL1 can affect brain functionality. For instance, in the subventricular zone, a recent report shows that *Jag1* neural-specific haploinsufficiency reduces the expansion of progenitor cells, which limits the production of migrating neuroblasts and olfactory bulb interneurons (Blackwood et al., 2020). In the adult hippocampus, Jag1 seems to be also the major Notch ligand controlling NSC maintenance and neurogenesis (Lavado and Oliver, 2014), and although *Dll1* is also expressed in this brain region, this appears to occur in mature neurons (Breunig et al., 2007). Therefore, any neurological dysfunction that relates to the production of new neurons in the postnatal dentate gyrus of *Dll1* haploinsufficient mice (e.g., memory acquisition; see below) could be a consequence of a misregulated developmental process (e.g., the one that caused a reduction in the number of SOX2^+^ cells).

Oligodendrocyte differentiation and myelination are processes occurring early after birth (Nishiyama et al., 2021). In agreement with premature OPC differentiation due to a reduced amount of DLL1, increased levels of myelination with the consequent reduction in OPCs were observed in brains of *Dll1*^+/lacZ^ mice during the early myelination waves. This pattern is similar to the one observed in brains of *Notch1* haploinsufficient mice (Givogri et al., 2002), suggesting that DLL1 is the main ligand interacting with NOTCH1 to control, at least, the initial waves of OPC differentiation and myelination. Interestingly, although alterations in myelination levels were not frequent in adult *Dll1*^+/lacZ^ mice, the absence of the transient increase in OPCs originated from the subventricular zone of juvenile WT mice suggests that myelin replacement dynamics and maintenance could be determined by Notch signaling and cause neurological alterations (see below).

Hydrocephalus is a congenital condition which origin is not completely understood. Generally, it is assumed that hydrocephalus originates from a disruption of the mechanisms that regulate the cerebrospinal fluid (CSF) homeostasis, including fluid production, absorption and circulation (Tully and Dobyns, 2014; McKnight et al., 2020). However, a recent report points to the possibility that altered developmental neurogenesis could cause hydrocephalus. Specifically, it has been found that mutations in *TRIM71* are frequent in congenital hydrocephalus patients (Duy et al., 2022). Interestingly, *Trim71* deficiency in mice causes premature neural differentiation and the development of small brains with fewer neurons than in WT brains (Duy et al., 2022), in close correlation with the observations in *Dll1* haploinsufficient mice. Detailed determinations in brain ventricles of *Trim71* deficient mice suggest a biomechanical disruption due to deficient neurogenesis as the initial origin of hydrocephalus (Duy et al., 2022). That is, scattered cell distribution, observed in brains of *Trim71* deficient and *Dll1* haploinsufficient mice, could reduce tissue stiffness to a point that is not sufficient to support the pressure from the CSF, causing dilation as ventricles adjust to a greater volume. Therefore, mild microcephaly resulting from deficient neurogenesis due to reduced DLL1 levels is a condition that favors hydrocephalus development.

### Attenuation of neurogenesis/oligodendrogenesis as a cause of neurological deficits

The genetic contribution to the morphological and behavioral phenotypes caused by *Dll1* haploinsufficiency cannot be discarded, but the fact that mice carrying the null *Dll1^lacZ^* allele are maintained as an outbred strain makes unlikely that a specific gene interaction is the cause of phenotypes observed. Rather, we propose that the display and penetrance of phenotypes observed could receive strong influence of environmental factors. Particularly relevant in this regard is hypoxia which is known to interact with Notch signaling (Landor and Lendahl, 2017) and has been shown to be determinant in scoliosis penetrance in association with reduced Notch signaling (Sparrow et al., 2012). Interestingly, the lack of Hif1a in brain results in alterations similar to the ones determined in the present study, such as reduced neurogenesis and hydrocephalus (Tomita et al., 2003). Therefore, reduced Notch signaling should behave as a sensitive condition to cause congenital brain abnormalities (e.g., microcephaly, hydrocephalus) due to environmental factors with possible impairment of relevant brain functions.

Although aging has not been studied in detail under *Dll1* haploinsufficiency, *Dll1*^+/lacZ^ mice have relatively high survival up to late adult life (i.e., 14 months of age) without evident neurological deficits within the adapted life in the animal facility. However, as shown here, specific tests revealed neurological deficits, some more penetrant than others, that might be part of the expected behavioral/psychiatric anomalies associated with microcephaly and/or hydrocephalus in humans (Tully and Dobyns, 2014; McKnight et al., 2020). Relevant in this regard, a recent report with human patients shows that DLL1 haploinsufficiency correlates with intellectual disability, autism and seizures, in addition to variable prenatally detected brain malformations (Fischer-Zirnsak et al., 2019). At this point, the specific cause of neurological deficits remains obscure; for instance, the inability to categorize objects was an interesting neurological impairment detected in *Dll1* haploinsufficient mice, but that could not be directly originated from disrupting adult neurogenesis in the hippocampus. Among possible causes, a reduced number of neurons could prevent correct neuronal circuit assembly, and circuit performance efficiency could be influenced by the morphological anomalies induced by ventriculomegaly and/or poor axonal myelination as it has been previously reported (Bonetto et al., 2021). In any case, the present work suggests that brain function disabilities in humans could arise from genetic or non-genetic factors (e.g., congenital) that reduce Notch signaling, which could be anticipated from early detection of mild microcephaly/hydrocephalus brain abnormalities. As a corollary, the efforts to identify the factors that interfere with Notch signaling (e.g., hypoxia) should result in the application of certain prenatal measures that likely prevent neurological deficits and increase life quality.

The mild alterations described here could be a factor that predisposes the development of specific neurological diseases, more easily to appreciate in those characterized by neuronal degeneration. Neuronal differentiation is not developmentally synchronized but, rather, different neuronal types emerge within particular regions of the developing brain and at a specific time. Therefore, in a genetic condition that prompts reduction in neurogenesis (e.g., *Dll1* haploinsufficiency), the transient influence of environmental factors (e.g., hypoxia) at a particular time would affect the production of a set of specific neurons. The number of neurons in the adult brain, determined after NPC proliferation/differentiation, neuroblast migration and neuronal circuit integration, defines the gap of neurons that can die. Hence, animals may be predisposed to neurological alterations even if the number of specific neuronal types in the adult brain are below normal, but above the threshold at which symptoms of a particular neurodegenerative disease are expressed or can be diagnosed. For example, fewer cholinergic neurons in the cortex or dopaminergic neurons in the substantia nigra are conditions that prompt early development of Alzheimer’s or Parkinson’s diseases, respectively (Spada, 2006; Oliveira et al., 2017). In a contrasting view, recently it was proposed that the function of a truncated form of Notch2, only present in humans (NOTCH2NL), is to enhance Notch signaling activation by DLL1 (Suzuki et al., 2018), hypothetically improving neurogenesis and cortex performance. Therefore, the present work has an additional translational value: treatments that boost Notch signaling could prevent neurodegeneration and improve intellectual performance in humans.

## Data availability statement

The raw data supporting the conclusions of this article will be made available by the authors, without undue reservation.

## Ethics statement

The animal study was reviewed and approved by the Ethics Committee of the Instituto de Biotecnología, Universidad Nacional Autónoma de México.

## Author contributions

LC led the research, designed experiments, analyzed the data, and wrote the manuscript. D-MA designed and performed the experiments, analyzed the data, and wrote the manuscript. CV, M-AD, EA-C, ED-S, GG-F, and MG-M designed and performed the experiments and analyzed the data.
